# The Outbreak of Yellow Fever

**Published:** 1888-10

**Authors:** 


### THE OUTBREAK OF YELLOW FEVER.

The outbreak of yellow fever in Florida has yet given but little indication of abatement. The lateness of the season, at its beginning, gave some hope that the reappearance of the disease might not be attended by such distress as characterized its occurrence on some former occasions, but that hope has not been realized, and the suffering, distress and demoralization have been great. Whilst there has been no general epidemic of it, yet the disease has not been confined even to the State of Florida.

Aside from the fatality and the suffering which have resulted from the disease, and the monetary loss which attends the interruption of commerce, three important facts have been made apparent by the recurrence of the disease this season: First, that the unsanitary conditions of many parts of our Southern States remain as in former years, notwithstanding the great advance in sanitary science in the last few years, and that those conditions, when accompanied by high thermometric and low barometric states, are favorable for the spread of the disease. Second, that so far the National quarantine laws have been inadequate, and have failed to prevent the importation of infected persons or property. Third, that notwithstanding the liberal provision made by the Government for investigation regarding the cause of the disease, no practical results of value have been attained, and that our ideas of its pathology and therapeutics are little, if any, in advance of what they were a quarter of a century ago.

If the disaster of this year should result in securing better sanitation in future in the Gulf States, and greater security for the inhabitants of them, the lesson of this year, calamitous as it has been, will not be without some compensating aspect, and some basis for hope for future immunity.

